# High-Elongation Starch Films by Hydroxypropylation Reaction with Low Glycerol Content

**DOI:** 10.1021/acsomega.5c04688

**Published:** 2025-10-01

**Authors:** Henrique Solowej Medeiros Lopes, Fernanda Andrade Tigre da Costa, Samir Leite Mathias, Cécile Bruzzèse Sillard, Alain Dufresne, Daniel Komatsu, Aparecido Junior de Menezes

**Affiliations:** † Federal University of São Carlos (UFSCar), 110 João Leme dos Santos Rd., Sorocaba, São Paulo 18052-780, Brazil; ‡ Technological College of Sorocaba (Fatec), 2015 Carlos Reinaldo Mendes Av., 2015, Sorocaba, São Paulo 18013-280, Brazil; § Nuclear and Energy Research Institute (IPEN-CNEN/SP), 2242 Prof. Lineu Prestes Av., São Paulo, São Paulo 05508-000, Brazil; ∥ CNRS (Grenoble INP, LGP2), Université Grenoble Alpes, Grenoble F38000, France; ⊥ Pontifical Catholic University of São Paulo (PUC), 290 Joubert Wey Street, Sorocaba, São Paulo 18030-070, Brazil

## Abstract

Hydroxypropylation is habitually used to modify starch by grafting bulky side groups onto its hydroxyls, significantly altering its structural, mechanical, and morphological properties. The hydroxypropylation reaction carried out in this study induced the gelatinization of starch, a phenomenon that is rarely observed in the literature due to the reaction conditions. This distinctive outcome is highly promising, as it enables the production of films with a reduced plasticizer content, offering a significant advantage over conventional methods. In this work, the identified presence of poly­(propylene oxide) (PPO) was investigated to enhance the flexibility of hydroxypropylated starch films, increasing elongation at break from 6% to 34% with only 7.5% glycerol (by weight of starch). PPO also accelerated retrogradation kinetics, attributed to its lubricating effect on starch molecules, and altered the surface energy, resulting in an increased polarity at lower molar ratios. These modifications led to films with reduced brittleness and improved mechanical performance. Scanning electron microscopy revealed a transition from brittle surfaces in native starch films to smoother, more ductile surfaces in modified films. This study demonstrates the effectiveness of PPO as a plasticizer, reducing the need for additional plasticizers and energy-intensive processing. These findings highlight the potential of hydroxypropylated starch films for sustainable packaging applications, advancing the development of eco-friendly materials with enhanced properties.

## Graphical abstract

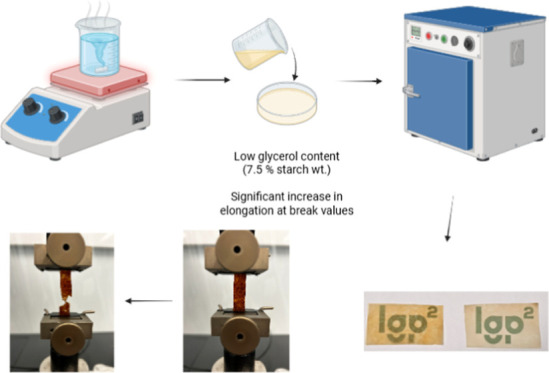

## 1 Introduction

Starch, a naturally occurring polysaccharide, is widely used in numerous industrial applications, mainly because of its biodegradability, low cost, and renewability. It is mainly composed of amylose and amylopectin, macromolecules composed of repetitive d-glucose monomers linked by α­(1,4) glycosidic bonding. Their molecular weight is approximately 10^6^ and 50–500 × 10^6^ g mol^–1^, respectively. Natively, amylose is linear and represents the amorphous portions of starch granule radial structure, with amylopectin being the semicrystalline phase with a branched double-helical structure. Greater quantities of amylose result in starch materials with higher elongation at break values.^1–4^ Starch-based materials often exhibit limitations such as poor mechanical properties, retrogradation, and susceptibility to water. Different approaches can be performed to enhance starch materials properties, such as altering starch characteristics by processing features,^5^ by obtention of starch nanoparticles,^6^ hybrid nanocomposites,^7^ and chemical modifications, such as hydroxypropylation or succinylation.^8–10^ The latter have been employed to address these limitations, enhancing the functionality and/or several properties of starch for a broader range of applications.^11–14^

Hydroxypropylation of starch involves the introduction of hydroxypropyl groups into the starch molecule.^15^ This modification is typically achieved through the reaction of starch with propylene oxide (PO) under alkaline conditions. Some factors influence the degree of substitution (DS) values, such as the reaction temperature, time, and molar ratio, playing crucial roles in determining the physicochemical properties of hydroxypropylated starch (HPS).^16^ The hydroxypropylation reaction proceeds via the nucleophilic attack of the hydroxyl groups of starch molecules and the epoxide ring of propylene oxide, resulting in the formation of ether linkages and thereby introducing hydroxypropyl groups onto the starch backbone. As a side reaction, poly­(propylene oxide) (PPO) can be formed, which is caused by excess of unreacted PO molecules in the bulk at specific temperatures and pressures, and by the presence of strong basis, mainly potassium hydroxide.^15–17^ This polymerization occurs via chain growth mechanism, where an adjacent hydroxyl group opens the epoxide ring of PO, forming a new hydroxyl species in the medium that can react with additional PO molecules present nearby, propagating through the chain.^17,18^

Starch granules can undergo disruption by a process called gelatinization, which eliminates all organized structures present in amylopectin branches, under high temperature and in the presence of a plasticizer.^2,19^ This process permits the production of a thermoplastic material, called thermoplastic starch (TPS), which presents interesting properties for the packaging industry, mainly biodegradability and renewability. However, the necessity of great quantities of a plasticizer to achieve adequate mechanical properties for the packaging industries reduces the biodegradability of TPS films, according to the literature,^20^ being greater when the quantity of plasticizers (like glycerol and/or sorbitol) in the TPS is lower. A study showed that up to 15% (starch wt) of plasticizer concentration presented 6–15% weight loss in soil, whereas higher concentrations of 30 and 45% (starch wt) presented no weight loss. Authors attributed this observation to the higher concentration of starch chains in the samples with lower amounts of plasticizer, which can be easily degraded by the soil and easily absorbed by the microorganisms present, as a carbon source, emphasizing the importance of decreasing the plasticizer content.^20^

In a previous work,^21^ hydroxypropylation of cassava starch resulted in a completely gelatinized material, asserted by the absence of starch granules under SEM analysis, added to the absence of typical crystalline peaks of starch structure under XRD analysis. Reaction features were fundamentally discussed, where a plasticized material was obtained caused by the formation of PPO, alkaline pre-treatment and temperature combinations, suggesting an innovative and distinct approach for obtaining films with decreased energy consumption and plasticizer content. Discussions regarding the film structure, extent of modification, thermal and mechanical properties, and morphology are provided.^21^ A distinctive feature observed was the formation of PPO molecules during the hydroxypropylation reaction and its effect within starch chains, lubricating the starch backbone and resulting in a plasticized material. This result, besides being innovative, showed promise for the obtention of films with higher flexibility and lower plasticizer content. Therefore, the objective of this current work is to produce and characterize films from gelatinized starch obtained straightforwardly by hydroxypropylation reaction using a significantly lower amount of plasticizer and lower temperatures of casting. The effect of PPO obtained in this work decreased drastically the quantity of plasticizers needed to obtain a high-elongation film, normally around 20–30% (starch wt) to 7.5% (starch wt), allied to a minor energy needed, producing cast films at significantly lower temperatures with high values of elongation at break.

## 2 Materials and Methods

### 2.1 Materials

Materials used were the following: food-grade cassava starch (Pinduca, Brazil), propylene oxide 99.5% (Sigma-Aldrich, USA), potassium hydroxide (Neon, Brazil), *n*-hexane 98.5% (Sigma-Aldrich, USA), glycerol anhydrous (Sigma-Aldrich, USA), ethanol 99%, diiodomethane 98% (Sigma-Aldrich, USA), deionized, and distilled water.

The reaction was carried out in a stainless-steel autoclave, electrically heated, and equipped with a manometer and a thermocouple to monitor the pressure and temperature.

### 2.2 Methods

The reaction was executed similarly to the method described in the literature,^8,21^ where starch was pretreated for 1 h with 50 mL of 40% potassium hydroxide (KOH) and ethanol/distilled water (5/1, v/v) solution, for the activation of hydroxyls groups, at a molar ratio of 1 [KOH/OH_starch_]. The mixture was then placed into the reactor with the addition of the desired amount of PO, according to the molar ratio defined, sealed, and heated to the desired temperature. Reaction takes place for 1 h, followed by cooling. After the reaction, the samples were washed with a solution of ethanol/distilled water to remove byproducts of the reaction, followed by 12 h of Soxhlet extraction with *n*-hexane and more 4 h of Soxhlet extraction with the same ethanol/distilled water solution mentioned previously. Samples’ designations are presented in Table 1.

**Table 1:** Experimental Design Levels in Parentheses and Sample Designation for Each Factor of Hydroxypropylated Starch Films

sample designation	temperature (°C)	molar ratio [PO/OH_starch_]
T115R04	115 (−1)	0.4 (−1)
T135R04	135 (+1)	0.4 (−1)
T115R08	115 (−1)	0.8 (+1)
T135R08	135 (+1)	0.8 (+1)

Starch was completely gelatinized during the reaction, as fundamentally discussed in a previous work,^21^ due to the grafting of bulkier side groups onto the starch backbone and/or the intermolecular presence of poly­(propylene oxide) chains, caused by the hydroxypropylation reaction carried out. In addition, the temperature above gelatinization and the alkaline pre-treatment may also have played a role.^200^ Therefore, the production of films was made via casting technique under gelatinization temperature, previously measured at 68 °C for the native starch used in this work.^21^ Hydroxypropylated starch (HPS) samples were solubilized in distilled water with a concentration of 15% (w/w) with 7.5% of glycerol (w/w starch-based) at 40 °C and magnetic stirring for 30 min and are designated according to Table 1. Control sample was prepared by solubilizing 3% (w/w) of native cassava starch and mixing with the same amount of glycerol at 70 °C under the same conditions and is designated as native starch throughout the manuscript. Films were dried in a ventilated oven during 24 h at 30 °C.

**Fourier-transform infrared spectroscopy (FTIR)** analyses were performed with a PerkinElmer Spectrum 100 instrument equipped with an attenuated total reflectance (ATR) apparatus. Films were analyzed from 4000 to 400 cm^–1^, with 16 scans and a resolution of 4 cm^–1^. **Thermogravimetry (TG)** was conducted from room temperature to 900 °C, under a heating rate of 10 °C·min^–1^ and oxygen atmosphere in a Mettler-Toledo TGA 2 system with up to 10 mg of the sample. **X-ray diffraction (XRD)** experiments were used to monitor film retrogradation using a PANalytical X’Pert Pro MPD from 5° to 50°, with a wavelength of 1.5418 Å at two distinct periods, 7 and 14 days after the film production. **Field Emission Gun Scanning Electron Microscopy (FEG-SEM)** was used to observe the cryogenically cut surfaces of HPS films with an FEI Quanta 250 FEG under 2.50 kV of tension. **Tensile** tests of films were performed using an Instron 5165 instrument under a 5 mm/min rate of grip separation. Samples of 50 mm in length and 10 mm in width were prepared according to ASTM D 882 ^201^ and tested at room temperature and humidity. Samples were conditioned in room temperature and relative humidity environment (around 20 °C and 60%, respectively) and thickness varied from 100 to 300 μm, approximately. Young’s modulus was calculated by drawing a tangent to the initial linear portion of the curve and dividing the corresponding stress per strain values within this region. Toe compensation was used to Hookean or No Hookean region when needed. An analysis of variance (ANOVA) and Tukey’s test were performed using Minitab, from a design of experiments (DOE) full factorial 2^2^. **Contact angle** measurements of drops of deionized water and diiodomethane applied on the surface of the films were measured on a DataPhysics OCA20 instrument under ambient conditions (20 °C and 20% relative humidity). Films were placed on the substrate. A 5 μL droplet of each liquid was dispensed from the syringe tip onto the sample surface and the contact angles after 2 s were monitored, and the average of at least three measurements was calculated. Table 2 shows the surface energy components of the test liquids according to refs 200–24.

**Table 2:** Polar and Dispersive Surface Energy Components of the Test Liquids

	γ_l_ (mJ m^–2^)	γ_l_^p^ (mJ m^–2^)	γ_l_^d^ (mJ m^–2^)
water	72.8	45.6	27.2
diiodomethane	50.8	0.0	50.8

The Fowkes method calculates the surface energy of a solid by combining the Young and Young–Dupré equations. This method separates the surface energy into dispersive and polar components. The process involves two steps: first, diiodomethane (with a negligible polar component) is used to determine the dispersive component of the solid’s surface energy. Subsequently, water (with a significant polar component) is employed to calculate the polar component of the solid’s surface energy. The underlying principle of the Fowkes theory is that the total surface energy is the sum of these dispersive and polar contributions, γ_s_ = γ_s_^p^ + γ_s_^d^.^200–24^

## 3 Results and Discussion

Figure 1 presents the infrared spectroscopy data for all modified (in color) and native starch (black) films. Considering the d-glucose repeating unit present in starch chains, the observation of new absorption bands at 1370–1360 cm^–1^ is related to bonding deformation of methyl groups (−CH_3_), being a distinctive feature after modification, i.e., absent in native starch. This is caused by the grafting and/or the presence of propylene oxide molecules in starch chains,^10,25^ where C–H bonding deforms, confirming the modification.

**Figure 1:**
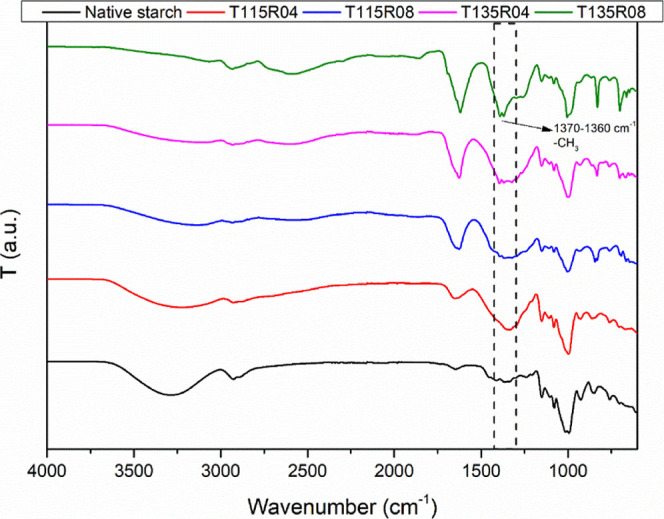
FTIR spectra for all modified and native starch film samples.

Remaining functional groups are hydroxyl groups (−OH), observed with some alterations in the regions of lower intensity around 3300 and 1650–1640 cm^–1^, probably due to the grafting of PO alongside the activation by KOH, and related to moisture.^26^ Ether moieties also presented a slight difference after modification, observed at 1100–1000 cm^–1^.^9,27^ Changes in hydrophobicity are expected, as mentioned in the literature,^8^ and may explain these alterations in hydroxyl absorption bands, observed between 3700 and 3000 cm^–1^, which was further investigated by contact angle measurements.

Figure 2 presents the thermo-oxidative degradation behavior for all samples. Dehydration of each sample after hydroxypropylation presented a different character, relative to the temperature of modification, as observed in Figure 2. Samples modified at lower temperatures (T115 pair) demanded higher temperatures to dehydrate, compared to native starch films, while T135 pair demanded lower temperatures. Similar features are also observed in Figure 1 (FTIR analysis), where higher temperatures of modification (T135 samples) presented more intense bands in the region of 1640–1650 cm^–1^ and less intense bands in the region of 3700–3000 cm^–1^, related to the water content and –OH groups, respectively. A different behavior is observed by other authors, where modified samples demanded higher temperatures to dehydrate, compared to native starch, which presented similar behavior here.^8^

**Figure 2:**
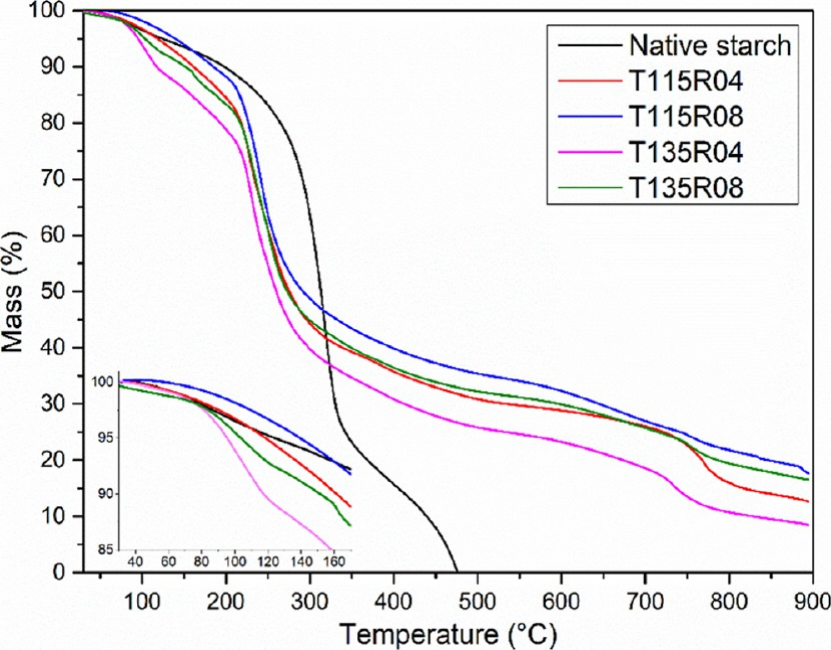
TG curves of all samples with emphasis on samples’ dehydration at the bottom left.

After dehydration of the samples, several steps of mass loss are observed for the modified samples and may be related to the decomposition of low-molar-mass molecules and volatiles, related to the presence of PPO and some unreacted PO, which was already detected by a low *T*_g_ observation in DSC analysis priorly^21^ and by the literature.^17^ The excess of PPO identified via TG, even after exhaustive Soxhlet extraction with *n*-hexane and ethanol/water solution, indicates a well-established interaction between the starch backbone and the PPO homopolymer. As discussed, what occurs parallel to the hydroxypropylation is the homopolymerization of propylene oxide molecules (PPO) by initiation and chain transfer, essentially discussed by ref 18 and further characterized by ref 17. These events may result in a high-molecular-weight branch of PPO covalently grafted on the starch backbone, somewhat near a TPS-*g*-PPO material, and also in the formation of low-molecular-weight molecules between starch chains, acting as a plasticizer, as discussed in ref 21.

In general, hydroxypropylation decreased thermal stability compared to the native starch film, as observed from the data reported in Table 3 (*D*_50%_). This behavior can be attributed to the above-mentioned plasticization effect, induced by PPO on starch chains when homopolymerized, alongside the presence of bulkier side groups of PO and its branches.^15,17^ Only the *D*_5%_ value of sample T115R08 presented a significantly higher temperature than the others and is related to the dehydration of the sample previously discussed. Notwithstanding, this same sample presented the most proximate decomposition temperatures to the native starch film. As discussed elsewhere,^21^ lower temperatures and higher molar ratios resulted in higher reaction yield (measured by mass gain), which may explain this behavior due to increased PO molecules grafting than PPO homopolymerization, the latter being partially removed by Soxhlet extraction. As long as starch is more plasticized, its thermal stability is decreased by the higher mobility of chains, and this may occur in our case by the presence of PPO within starch chains. Commercial PPO with high-molar-mass presents thermo-oxidative degradation near 250 °C,^28^ similar to starch materials, and may be overlapped in this case, which explains lower values obtained for *D*_15%_ and *D*_50%_ compared to native starch. Residual mass is observed at 900 °C for the modified samples and is related to the presence of residual potassium species, also observed by others with different inorganic particles.^29^ The lower the molar ratio, the lower the residual mass, which may indicate that potassium could also be complexed with some PPO molecules. In addition, due to the harsh conditions of the analysis, native starch showed negligible residual mass.

**Table 3:** Decomposition Temperatures of Each Sample Analyzed by TG^a^

sample	*D*_5%_ (°C)	*D*_15%_ (°C)	*D*_50%_ (°C)	*R*_900°C_ (%)
native	124.12	239.39	313.08	
T115R04	118.55	196.77	275.62	12.64
T115R08	139.36	215.94	291.69	17.63
T135R04	95.22	158.48	260.02	8.50
T135R08	103.65	187.82	272.81	16.55

a *D* = decomposition. *R* = residue.

Figure 3 presents the XRD data after 7 (straight lines) and 13 (dotted lines) days of film casting.

**Figure 3:**
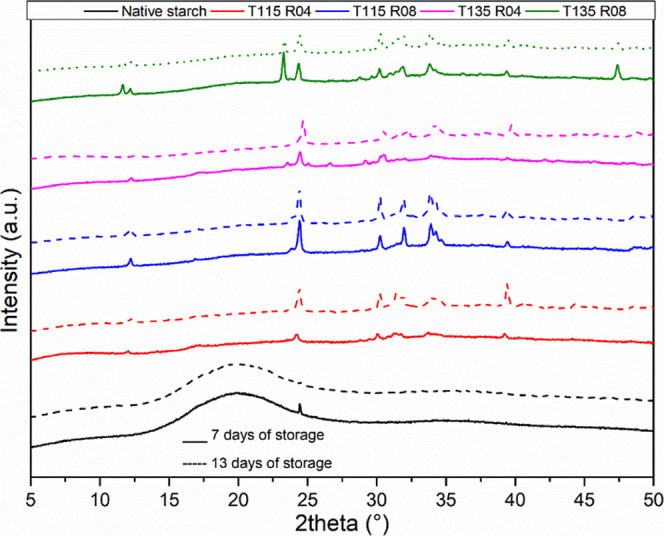
XRD data of all films.

Native starch presented an anomalous behavior after storage with a reduction of the peak related to the V_H_ structure. This structure consists in single-helical-arranged amylose chains.^4^ Modified starch samples present several crystalline peaks in the region near 12° and 23.5°, which are related to the fast crystallization of amylose into V_H_ structure (single-helix crystals), according to the description of refs 2, 4, and 19 . The plasticization of starch chains by PPO facilitates even more the crystallization of amylose into this structure over aging, clearly observed for samples with a higher molar ratio and temperature. In addition, short-range structures were already identified via FTIR in a previous work,^21^ consisting in local ordered structures of double-helical amylose chains also and strongly corroborating with the accelerated retrogradation kinetics.

Similar behavior was observed by Lafargue and collaborators during the drying of cast films from hydroxypropylated starch, where several B-type structures were detected by XRD analysis in a short period.^11^ This structure consists of amylopectin double-helical chains arrangement packed in a hexagonal unit cell with the presence of water molecules,^2,4,30^ not observed here. Chuenkamol et al. also observed increased retrograded structures in hydroxypropylated canna starch.^13^ These observations contradict the report of refs 31–33 , where authors obtained a reduction in starch retrogradation kinetics after hydroxypropylation. It is worth noticing that in these latter works, hydroxypropylation was not performed under high temperature and high pressure and starch was not gelatinized by the reaction.

Slowing retrogradation kinetics is expected during the grafting of bulkier side groups in starch chains by hydroxypropylation, preventing chains from reassociating by increased mobility and/or hydrogen bonding. Nevertheless, in our case, the presence of PPO facilitated amylose mobility and explains the results obtained.^32,34^ On the other hand, amylopectin chains seem not to be influenced by the plasticization effect, by the absence of characteristic peaks, possibly related to its branched and less organized structure.

Peaks observed at 30–35° and 40° regions are related to the presence of potassium compounds, such as KOH, K_2_CO_3_, and βK_2_O, as identified by Díaz-Terán.^35^ These compounds were not solubilized during the Soxhlet extraction with ethanol/distilled water nor *n*-hexane and are formed due to potassium interactions with –CH_2_ groups, also observed as residues from TG analysis and as particles in SEM micrographs. Potassium compounds are usually detected at these regions, according to the literature.^36–39^

Figure 4 shows all of the micrographs obtained via SEM analysis of fractured surface films from modified and native starch.

**Figure 4:**
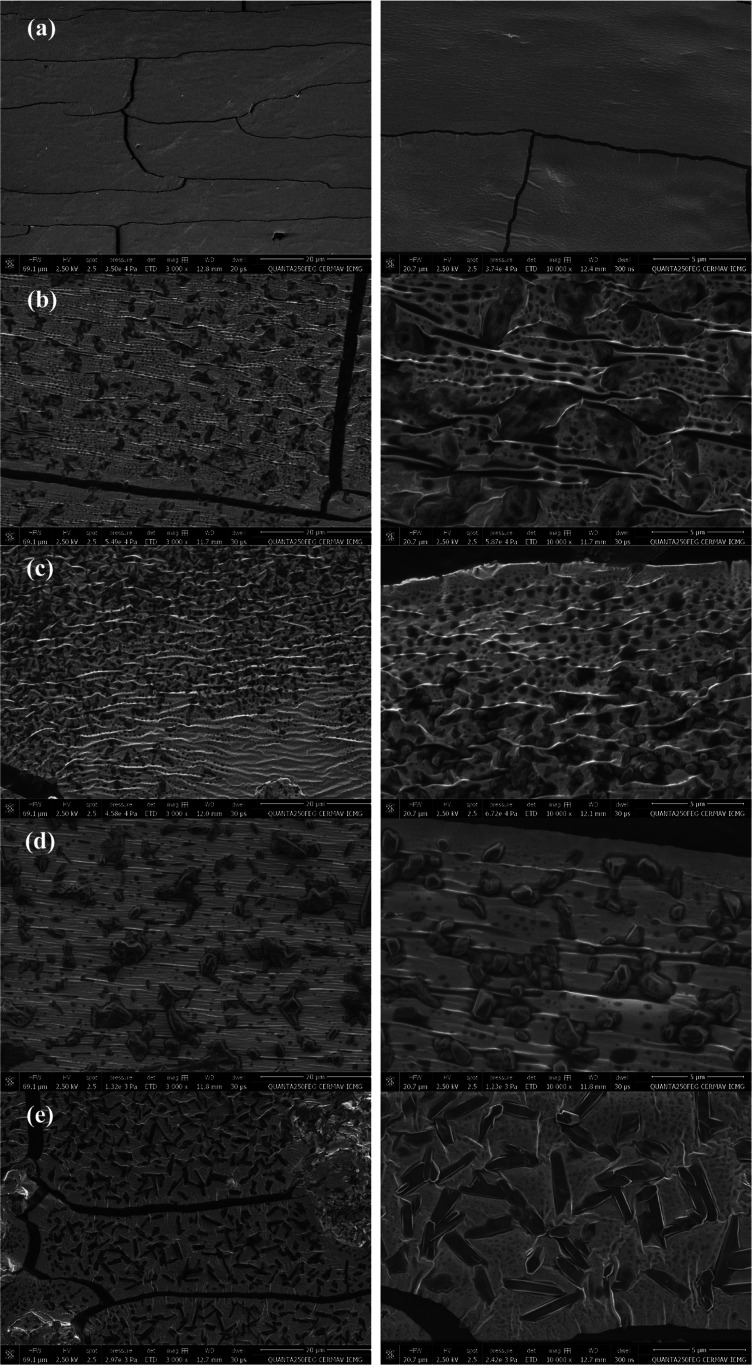
SEM micrographs of all samples’ fractured surfaces with 3000× of magnification (left column) and 10,000× of magnification (right column). Native starch (a), T115R04 (b), T115R08 (c), T135R04 (d), and T135R08 (e) films.

Native starch film presented a smooth surface with the presence of cracks, as is clearly seen in Figure 4 (a). A rougher surface is observed for samples modified at lower temperatures (b and c), compared to others of higher temperatures (d and e). Cracks are also present in modified samples, but they seem to be less frequent. These observations can be attributed to a more brittle behavior.^5^ Additionally, all samples presented a great quantity of potassium compounds with different morphologies, even after extensive Soxhlet extraction with ethanol/distilled water and *n*-hexane, underlining a possible interaction with starch’s activated hydroxyls. Similar observations were made by others with different substrates.^37,38^ The presence of these particles results in a high quantity of porosity and defects on the fractured surface, which is observed in all modified samples. Cavities due to potassium particles are observed, surrounded by tiny holes. These observations differ from the observed ones by refs 40 and 41 during casting of hydroxypropylated films due to the absence of potassium.

Figure 5 contains the tensile strength and elongation at break values obtained from tensile tests for all modified samples and native starch. Typical tensile strength curves are available in the Supporting Information (Figure S1).

**Figure 5:**
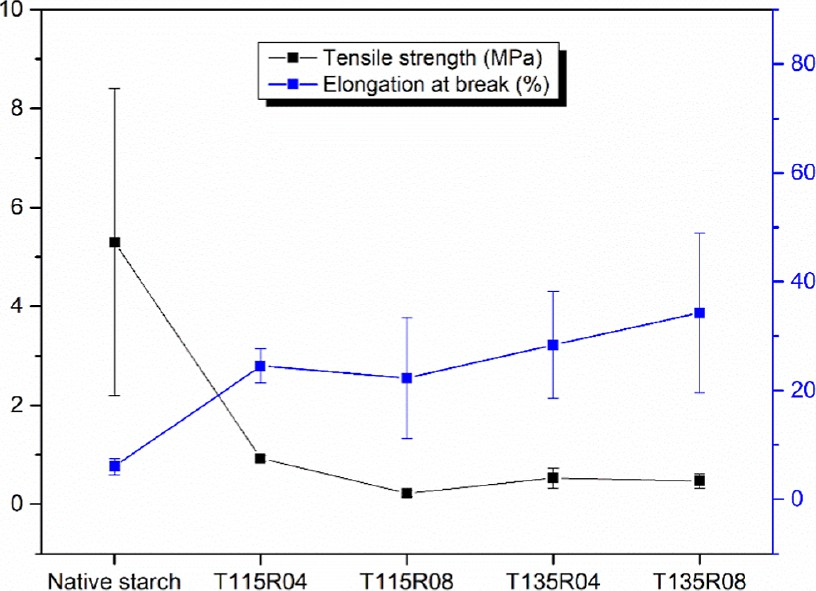
Tensile strength (left axis) and elongation at break (right axis) of all samples.

Here, the plasticization effect performed during hydroxypropylation is evidenced. In detriment of tensile strength values, all modified samples presented an increase in the elongation at break. It also should be considered that the presence of potassium compounds must have been playing a negative role in the sample’s mechanical properties. As noticed from the discussions above, the presence of PPO molecules facilitates mobility, explaining this observation. Lafargue et al. obtained hydroxypropylated starch films with significantly lower elongation at break, of 7.5–9%, considering that the reaction performed by the authors did not disrupt native starch granules.^11^ On the other hand, Chaudhary and collaborators obtained less rigid starch material when hydroxypropylated. The latter authors mentioned that the grafting of PO molecules within starch molecules prevents chain association, resulting in a more plasticized material. This behavior may have been maximized here, by a great homopolymerization of PO, where for maximum stress values, modified starch presented lower values, and at elongation at break values, for which modified starch samples presented values twice as high as those of the unmodified sample. Vorwerg et al. obtained also a more rigid hydroxypropylated starch film, with elongation at break values up to 24% and significantly higher maximum stress for the films.^14,42^ Table 4 summarizes the mechanical properties obtained. The lower value of tensile strength and Young’s modulus presented by sample T115R08 is clearly related to its rougher morphology, observed by SEM (Figure 4c) when compared mainly to T115R04, with higher stiffness, e.g., higher Young’s modulus.

**Table 4:** Mechanical Properties Obtained for All Samples

sample	tensile strength (MPa)^a^	elongation at break (%)^a^	Young’s modulus (MPa)	DS (from ref 21)
native starch	5.28 ± 3.10	6.02 ± 1.51	147.84 ± 51.92	
T115R04	0.93 ± 0.06^aa^	24.52 ± 3.18^aa^	52.55 ± 16.60^aa^	0.15
T115R08	0.22 ± 0.07^ab^	22.29 ± 11.14^aa^	15.71 ± 1.28^aa^	0.45
T135R04	0.53 ± 0.19^aa^	28.37 ± 9.76^aa^	13.73 ± 2.82^aa^	0.10
T135R08	0.47 ± 0.15^ab^	34.24 ± 14.73^aa^	17.57 ± 5.81^aa^	0.28

a Different lowercase letters indicate a different grouping under Tukey’s test. The first letter corresponds to Tukey’s test for temperature, and the second letter corresponds to Tukey’s test for the molar ratio.

Modified samples are probably under low starch–starch interactions due to the presence of PPO molecules intermolecularly or grafted, hindering hydrogen interactions between starch chain and even lubricating amylopectin entanglements, improving mobility.^34^ Nevertheless, obtained mechanical properties of this work are similar or higher to those reported by ref 5, where blends of 80/20 TPS/PBAT, also conditioned under high relative humidity and obtained by extrusion and related processes, were tested and presented values of 20–30%, 0.2–2 MPa, and ∼2.5 MPa of elongation at break, tensile strength, and Young’s modulus, respectively, evidencing the plasticization effect of pure hydroxypropylated starch/glycerol films in comparison to blended TPS materials obtained by melting processing. It is worth noticing that the glycerol content used in the mentioned work was much higher when compared to here, and the effect of relative humidity plays a role in starch’s plasticization, lubricating chains. It is also higher than that observed by ref 43, where hydroxypropylated high-amylose starch was prepared with different quantities and types of plasticizers. Authors obtained elongation at break values around 18% at a plasticizer concentration of 15% (w/w).

As available in the Supporting Information file (Figure S2), ^13^C NMR analysis revealed the successful introduction of methyl groups in all modified samples, confirming the extent of modification, essentially discussed elsewhere.^21^ A new signal at 20 ppm region was observed for all modified samples, related to CH_3_ introduced by PO through ether linkages into the starch backbone, most likely at C2 and C3, also observed by others.^40,44–46^ Values of DS were estimated by the ratio of the methyl signal and C1 and are shown in Table 4. It was mentioned that a clear correlation between DS values and the parameters of modification exists; that is, higher molar ratios led to increased DS (0.45 and 0.28 for temperatures of 115 and 135 °C, respectively), when compared to lower molar ratios (0.15 and 0.10 for temperatures of 115 and 135 °C, respectively), feature inversely observed for the temperature.

An interesting relation between the degree of substitution and the obtained mechanical properties was observed and is shown in Figure 6.

**Figure 6:**
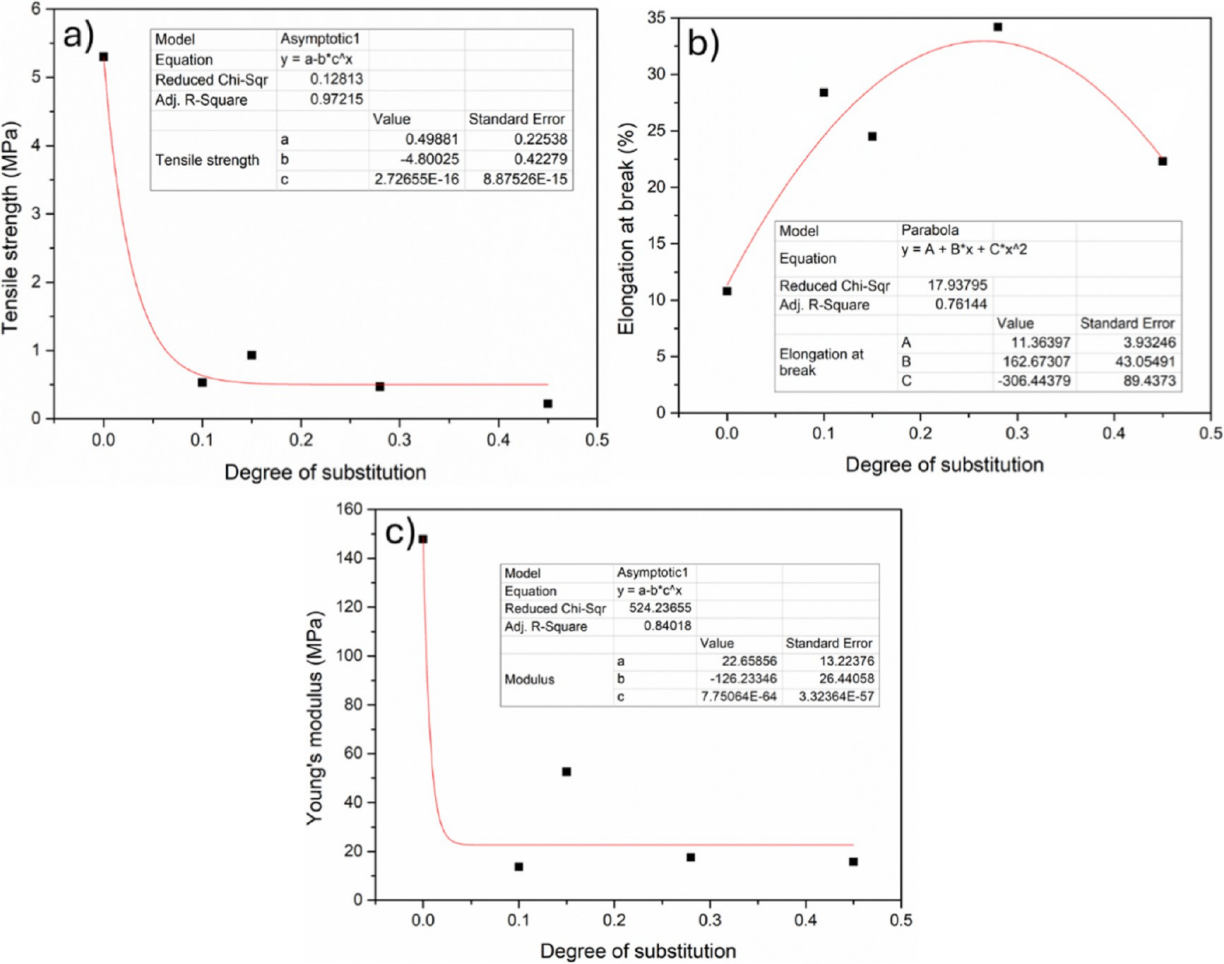
Nonlinear fit behavior observed for DS and mechanical properties of (a) tensile strength, (b) elongation at break, and (c) Young’s modulus.

A nonlinear relationship is observed between the obtained DS and the mechanical properties. For tensile strength, an exponential decrease in its values is observed for all DS values obtained, evidencing the plasticizing effect of PPO. Similar behavior but less pronounced is observed for Young’s modulus. Otherwise, for elongation at break, a polynomial relationship is observed, in the form of a parabola, suggesting the existence of an optimal point of modification to optimize the elongation at break property. These observations emphasize the importance of controlling the molar ratio during the modification reaction, which is directly related to the grafting and/or formation of PPO homopolymers in the bulk, affecting the mechanical properties obtained.

As shown in Table 5, the molar ratio presented a *P*-value under 0.05, using one-way ANOVA interactions, confirming the above-mentioned discussion about the molar ratio influence in tensile strength, alongside a high *F*-value, the opposite observed to the temperature, followed by different groupings under Tukey’s test (see Table 4), evidencing the significant effect. Elongation at break and Young’s modulus had no influence on this analysis, presenting a *P*-value higher than 0.05 and the results are not shown, which is similar to the one observed under Tukey’s test, where the same grouping was obtained for all conditions.

**Table 5:** *F* and *P* Value of Each Parameter Analyzed Obtained under ANOVA with Tensile Strength as Output

source	*F*-value	*P*-value
temperature	0.02	0.902
molar ratio	11.69	0.007

The contact angle data presented in Tables 6 and 7 reveal that hydroxypropylated starch exhibits a lower water contact angle compared to native starch, indicating an increased affinity for polar solvents.

**Table 6:** Samples’ Contact Angle on Water and Diiodomethane

	contact angle (deg)	
samples	water	diiodomethane
native starch	60.25 ± 0.75	50.85 ± 0.51
T115R04	25.13 ± 0.86	60.90 ± 0.06
T115R08	29.86 ± 0.46	40.76 ± 0.10
T135R04	28.17 ± 0.65	56.54 ± 0.32
T135R08	49.88 ± 1.62	39.05 ± 0.10

**Table 7:** Samples’ Surface Energy Calculated through the Fowkes Method

samples	γ_s_^p^ (mJ m^–2^)	γ_s_^d^ (mJ m^–2^)	γ_s_ (mJ m^–2^)
native starch	12.78 ± 0.46	33.80 ± 0.29	46.58 ± 0.54
T115R04	38.19 ± 0.43	28.06 ± 0.04	66.24 ± 0.43
T115R08	15.80 ± 0.23	39.23 ± 0.05	55.02 ± 0.23
T135R04	34.48 ± 0.37	30.56 ± 0.18	65.05 ± 0.41
T135R08	27.33 ± 0.93	40.09 ± 0.05	67.42 ± 0.93

The temperature and molar ratio can inflict some variations to these values, showing an inverse relation among them, where the lower the temperature and molar ratio are, the greater the affinity for polar solvents. A direct relationship can be attributed to nonpolar solvents, where the lower the temperature and molar ratio, the lower the affinity for nonpolar solvents. Jonhed et al. observed that the addition of glycerol also decreased the contact angle values for hydroxypropylated starch films,^47^ while Zhang et al. (2013, a,b) obtained a hydrophilic behavior for hydroxypropyl starch through water contact angle measurements, but with higher values compared to this work (72.3° and 84.3°).^41,48^ Both authors mentioned that this is an important behavior when water solubility is desired, a feature observed here.

High molar ratios result in higher modification rates, which produce more PPO molecules, as mentioned in ref 17 and observed previously through high mass gain values obtained,^21^ and this decreases γ_s_^p^ values among modified samples, as observed in Table 6, by increasing the apolar component. A less marked trend is observed for low-molar-ratio samples, where the homopolymerization of PPO is probably lower. Nevertheless, all samples presented hydrophilic behavior and deviate from the findings of refs 8 and 9 , possibly due to the plasticizing effect of residual glycerol and PPO within the modified starch, as mentioned by refs 49 and 50 , and the presence of residual KOH, which must have been taken into account. Increased γ_s_^d^ was also obtained by ref 50 and was related to surface roughness. Increased surface energy for hydroxypropylated starch coatings is discussed in ref 51, and trends, similar to those observed in this work, may be an interesting feature for packaging purposes.

In addition, HPS films presented a yellowish coloration compared to the native starch film, with good transparency and mechanical integrity, as can be seen in Figure 7.

**Figure 7:**
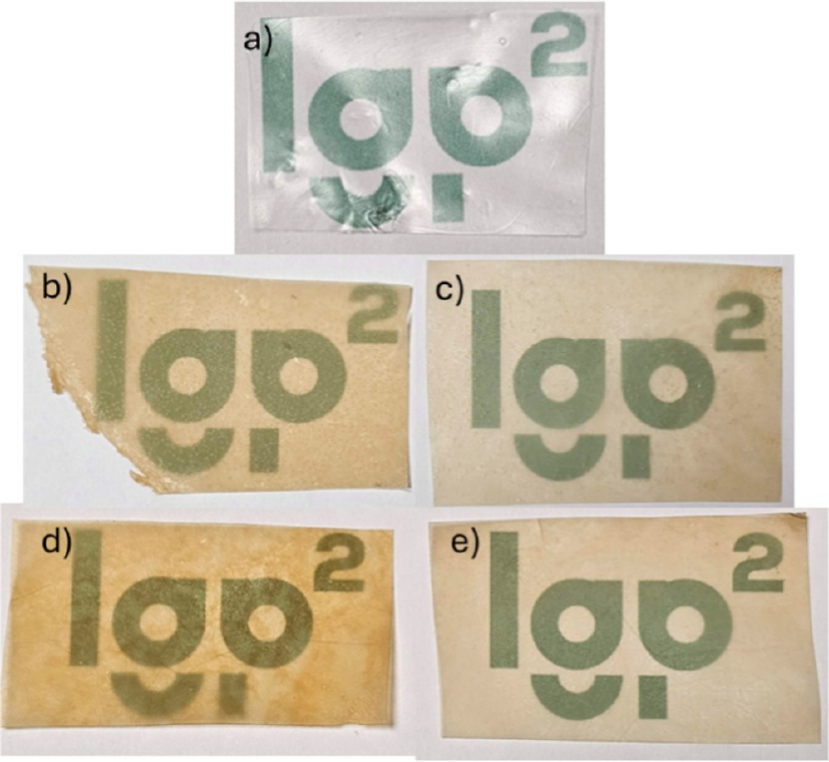
Films of native starch (a) and modified starch obtained in this work. Designation: T115R04 (b), T115R08 (c), T135R04 (d), T135R08 (e).

The yellowish coloration seems to be more pronounced in samples modified under lower molar ratios (0.4), as seen in Figure 7b,d, especially sample T135R04. Curiously, both samples presented the lowest DS values, indicating that the extent of modification may not have caused this. White, transparent films are observed for this type of material.^43^ However, the reaction temperature may have produced the yellowish aspect due to oxidative decomposition, despite the absence of carbonyl groups in FTIR analysis. It was also observed for high-pH hydroxypropylated starch materials.^25^

Results obtained in this work emphasize the important effect that PPO had in plasticizing HPS chains, with a significant decrease in glycerol content, maintaining the film characteristic with significantly improved flexibility, when compared to starch-based materials in the literature.^21,43^ Features observed are promising for further investigations regarding reaction parameters, with the intent to control the amount of PPO molecules, which clearly altered the properties obtained.

## 4 Conclusion

In this study, the plasticization effect of poly­(propylene oxide) (PPO) on hydroxypropylated starch films was thoroughly investigated, revealing significant enhancements in material properties. FTIR analyses confirmed the successful integration of the modification by identifying characteristic CH_3_ groups. TG highlighted the presence of PPO, that further contributed to the observed plasticization. XRD analysis showed accelerated retrogradation kinetics, ascribed to PPO’s lubricating effect on HPS molecules, and SEM revealed that native starch films were brittle, while the modified films exhibited improved flexibility. Mechanical testing corroborated these findings, with the modified films displaying significantly higher elongation at break values, from 6 to 34%, indicating enhanced plasticization and showing correlation with DS values obtained previously. Contact angle showed that hydroxypropylation increased the surface energy of all samples, which may be desired for packaging purposes, also denoting the presence of PPO and its influence on the results obtained. Remaining challenges respective to this study are related to biodegradability and toxicity assessment, highlighting the significance of using films from hydroxypropylated starch for food to industrial applications, aiming at an environmentally friendly discard route, controlling the formation of PPO, seeking mechanical property tuning, and developing a more effective washing procedure for potassium compounds excess, which definitely played a role in the films' properties. This study deals with how PPO modifies hydroxypropylated starch films, changing their mechanical properties to a high elongation material, thus offering interesting features for use as eco-friendly packaging, such as lower temperature casting production and significant lower glycerol content.

## Supplementary Material



## Notes

Typical curves of stress per strain and ^13^C NMR (PDF)
