# Supplementary material for: High-Elongation Starch Films by Hydroxypropylation Reaction with Low Glycerol Content
Source: ACS Omega. 2025 Oct 1;10(40):46822–31. doi: 10.1021/acsomega.5c04688 (PMC12529193; doi:10.1021/acsomega.5c04688)
Supplement: Supplementary file 1 [file ao5c04688_si_001.pdf]

HIGH-ELONGATION STARCH FILMS BY HYDROXYPROPYLATION REACTION WITH LOW GLYCEROL CONTENT

Henrique Solowej Medeiros Lopes[1,2,4], Fernanda Andrade Tigre da Costa[3,4], Samir Leite Mathias[1,4], Cécile Bruzzèse Sillard[4], Alain Dufresne[4], Daniel Komatsu[2,5] Aparecido Junior de Menezes[1*]

[1]Federal University of São Carlos (UFSCar), 110 João Leme dos Santos Rd., Sorocaba, SP, 18052-780, Brazil

[2]Technological College of Sorocaba (Fatec), 2015 Carlos Reinaldo Mendes Av., 2015, Sorocaba, SP, 18013-280, Brazil

[3]Nuclear and Energy Research Institute (IPEN-CNEN/SP), 2242 Prof. Lineu Prestes Av., São Paulo, SP, Brazil

[4]Univ. Grenoble Alpes, CNRS (Grenoble INP, LGP2, F38000, Grenoble), France

[5]Pontifical Catholic University of São Paulo (PUC), 290 Joubert Wey St., Sorocaba, SP, 18030-070, Brazil

*e-mail: jrmenezes@ufscar.br

Supporting information

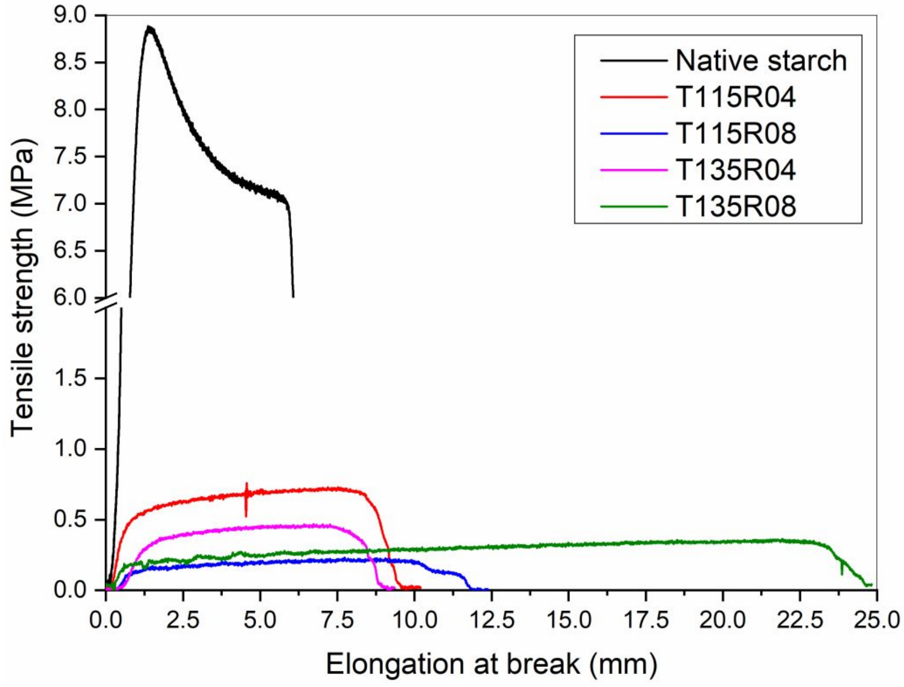

Figure S1. Typical curves of tensile strength per elongation at break of tensile tests performed.

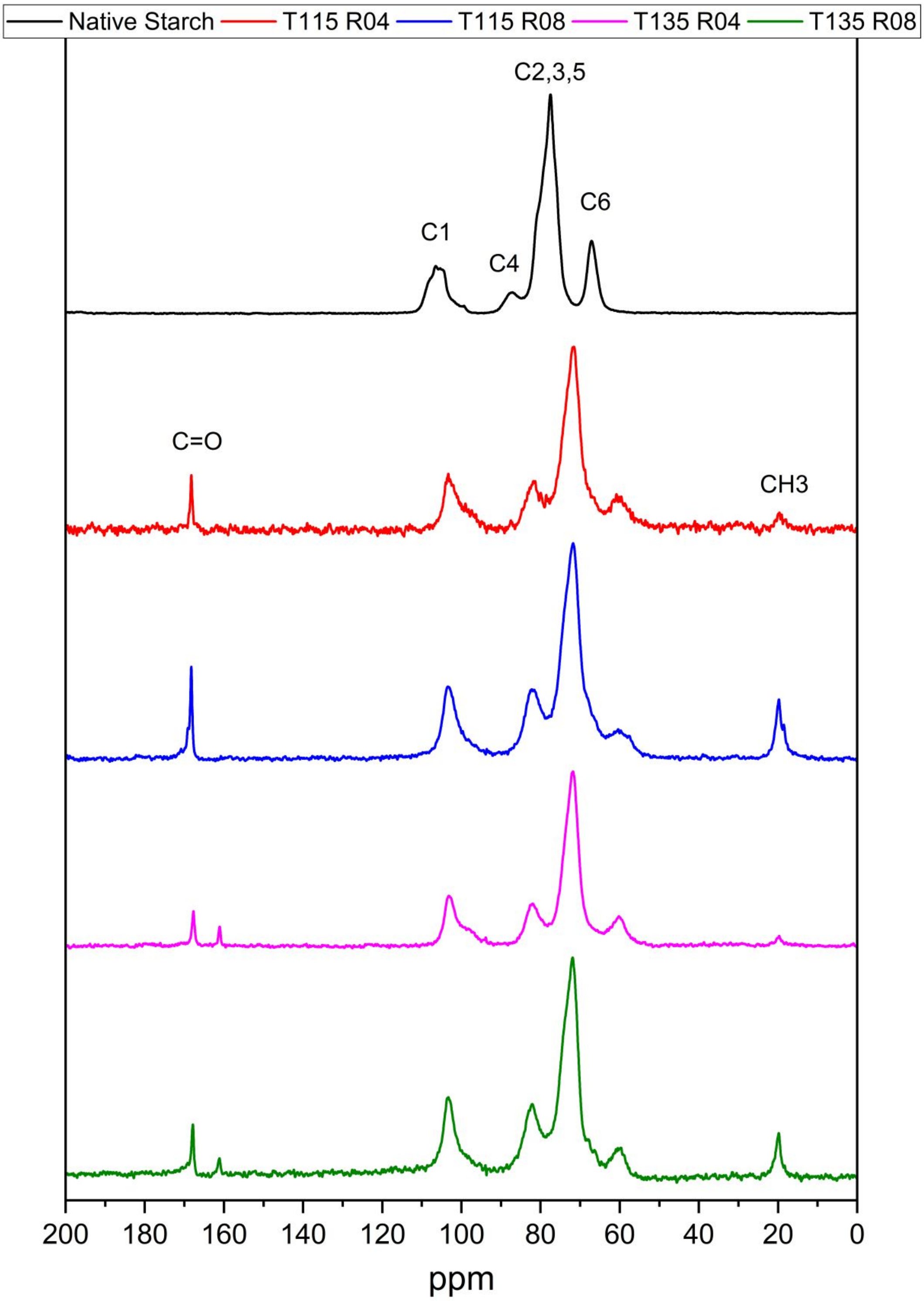

Figure S2. ^{13}C-NMR spectra with signal assignment for native starch and modified samples. Reprinted with permission from Lopes et al. Gelatinized Cassava Starch Obtained via Low Molar Ratio Hydroxypropylation Reaction. ACS Omega 2025 10 (12), 12543-12552. DOI: 10.1021/acsomega.5c00246. Copyright 2025, American Chemical Society (ACS).
